# Primary Segmental Volvulus of Small Intestine: Surgical Perspectives According to Age at Diagnosis

**DOI:** 10.3389/fped.2019.00146

**Published:** 2019-04-17

**Authors:** Soo-Hong Kim, Yong-Hoon Cho, Hae-Young Kim

**Affiliations:** ^1^Department of Surgery, Pusan National University Yangsan Hospital, Yangsan, South Korea; ^2^Research Institute for Convergence of Biomedical Science and Technology, Pusan National University Yangsan Hospital, Pusan National University School of Medicine, Yangsan, South Korea

**Keywords:** primary, segmental, volvulus, small intestine, postnatal

## Abstract

**Background:** Small intestinal volvulus often occurs with malrotation. However, in some cases, it could develop without any other clinical conditions, and this is called primary segmental volvulus (PSV) of the small intestine. Two types of PSV (early and late neonatal) have been described previously, especially in preterms. Moreover, there were other cases occurring beyond the neonatal period.

**Methods:** The medical records of 14 cases definitively identified as PSV were retrospectively reviewed. The patients were divided into 2 groups according to postnatal age at diagnosis: neonatal group and beyond neonatal group. Then, the 2 groups were compared in terms of clinical features.

**Results:** There were 11 patients in the neonatal group (78.6%) and 3 patients in the beyond neonatal group (21.4%). There were no differences in gestational age, birth weight, and ratio of prematurity. In the neonatal group, the antenatal abnormal sonographic findings were found more frequently and the perforation of the involved segment were relatively common. Meanwhile, the involved segment was confined to the ileum and more commonly associated with mesentery change in the beyond neonatal group. There was no mortality.

**Conclusion:** The 2 clinical types of PSV according to postnatal age at diagnosis show some differences in clinical features. Moreover, PSV should be considered a possible cause of surgical problems beyond the neonatal period.

## Introduction

Most cases of intestinal volvulus are identified during the neonatal period in association with the congenital anomaly of midgut rotation (1, 2). Other forms of volvulus have been described in association with certain clinical conditions, such as internal hernia, inter-loop adhesive band, mesenteric defects, and mass lesions, and it may occur at any age (3–6). However, intestinal segmental volvulus could develop without malrotation or any other associated diseases. This condition could be considered a primary segmental volvulus (PSV) and characterized as the abnormal twisting of the involved bowel around its own mesentery (7).

There are few studies about PSV, which are usually small case series or sporadic case reports (1, 2, 8, 9). Therefore, little information about PSV is available. Additionally, previous studies have focused on neonatal cases, especially in premature infants (1, 8–13). Therefore, it is unusual to find cases of PSV occurring beyond the neonatal period (2). Considering its clinical characteristics, including its possibility to rapidly progress to fatal outcomes without prompt management and to occur at any age, it is important to be more concerned about the clinical features of PSV.

In this study, we analyzed the clinical characteristics of PSV according to postnatal age at occurrence (neonatal period or beyond the neonatal period), with the expectation that identifying its clinical implication can aid in patient management.

## Methods

### Subjects

A total of 19 cases of segmental volvulus were postoperatively confirmed between January 1999 and December 2017. The diagnosis of PSV was based on operative findings, and 14 cases were finally identified. The other 5 cases were associated with intestinal atresia, band, tumor lesion, Meckel's diverticulum, and other predisposing intra-abdominal diseases, and were excluded from this study. This study was approved by the Pusan National University Yangsan Hospital Institutional Review Board (IRB No. L-2018-138) and carried out in accordance with the recommendations of IRB committee.

### Analysis of Clinical Features

Medical records were reviewed retrospectively for the patients' demographics, gestational data, antenatal and postnatal clinical findings, preoperative diagnosis, operative findings and procedures, pathologic results, and complications and outcomes of the treatment.

According to postnatal age at operation, we divided the patients into 2 groups: group A (neonatal group, in which PSV was diagnosed during the neonatal period) and group B (beyond neonatal group, in which had PSV was diagnosed after the neonatal period). The clinical characteristics were compared between the 2 groups.

The chi-square test, Kruskal-Wallis test, and Mann-Whitney *U*-test were used with SPSS software (ver. 21.0; SPSS Inc., Chicago, IL, USA) for statistical analysis. A *p* < 0.05 was considered statistically significant.

## Results

Among the patients, 11 were classified into group A (78.6%) and 3 in group B (21.4%).

### Demographic Findings (Table 1)

The male to female ratio, gestational age and birth weight were not significantly different between both groups. During antenatal care, abnormal sonographic findings were more frequently found in group A patients (*p* = 0.012), meanwhile, there were no antenatal findings in group B. A dilated bowel loop was most frequently observed finding (9 patients) and polyhydroamniosis (2 patients), intrauterine growth retardation were also accompanied, each. Cesarean-section delivery was more common than normal vaginal delivery in both groups but no statistical difference was observed.

**Table 1:** Demographic findings.

	**Group A (*n* = 11)**	**Group B (*n* = 3)**	***p*-value**
Gender [M: F (%)]	8:3 (72.7)	1:2 (33.3)	0.365
Gestational age (weeks)	36.7 ± 3.6 (30~41)	35.7 ± 5.9 (29~40)	0.885
Preterm: Fullterm (%)	4 (36.3)	1:2 (33.3)	0.717
Birth weight (g)	3,022.7 ± 797.4 (1,570~4,110)	2,727.3 ± 1,489.9 (1,012~3,770)	0.938
Antenatal abnormal sonographic findings [*n* (%)]	9 (81.8)	0 (0.0)	0.012
Delivery [NSVD: CS (%)]	3:8 (27.2)	1:2 (66.7)	0.530
Maternal age (years)	29.5 ± 4.6 (24~38)	32 ± 5.8 (27~41)	0.445
Age at operation (days)	3.0 ± 2.2 (0~7)	417.0 ± 599.9 (66~1,110)	0.005
Main presentations [*n* (%)]			
Abdominal distention	7 (63.6)	2 (66.7)	0.843
Bilous vomiting	4 (36.3)	1 (33.3)	0.925
Others	1 (9.1)	1 (33.3)	0.305
Preoperative diagnosis [*n* (%)]			0.103
Intestinal atresia	6 (54.5)	0 (0.0)	
Bowel perforation	3 (27.2)	1 (33.3)	
Midgut volvulus	0 (0.0)	1 (33.3)	
Others	2 (18.1)	1 (33.3)	

*NSVD, normal spontaneous vaginal delivery; CS, cesarean section. Values are presented as mean ± standard deviation or patient number*.

The main clinical feature was non-specific abdominal distention in both groups. Other common clinical findings were bilious vomiting in group A and pain in group B. Additionally, 1 case in group B was an incidental diagnosis during surgical exploration for biliary atresia. Intestinal atresia was the most common preoperative diagnosis in group A, and followed by bowel perforation including meconium peritonitis, necrotizing enterocolitis. Meconium-related ileus, Hirschsprung's disease were also suspected. In group B, the preoperative diagnosis included necrotizing enterocolitis and midgut volvulus.

### Operative Findings and Outcomes (Table 2)

In both groups, the site of volvulus was most commonly the distal small intestine (ileum), 81.8% in group A and 100.0% in group B. Although grossly necrotic change of the involved segment was identified in all cases of group A and in 66.7% of group B, perforation of the intestine was found only in group A (54.5%) (*p* = 0.050). Meanwhile, most cases were not associated with any other conditions, and a suspicious predisposing factor (a long and narrow mesentery of involved segment) was identified in only 2 cases of group B (2 of 14, 14.3%).

**Table 2:** Operative findings and outcomes.

	**Group A (*n* = 11)**	**Group B (*n* = 3)**	***p*-value**
Site of volvulus [*n* (%)]			0.112
Jejunum	2 (18.1)	0 (0.0)	
Proximal ileum	0 (0.0)	2 (66.7)	
Distal ileum	9 (81.8)	1 (33.3)	
Perforation [*n* (%)]	6 (54.5)	0 (0.0)	0.050
Necrosis [*n* (%)]	11 (100.0)	2 (66.7)	0.305
Operation procedure [*n* (%)]			0.365
SR with ileostomy	7 (63.6)	1 (33.3)	
SR with PA	4 (36.3)	1 (33.3)	
Manual detorsion	0 (0.0)	1 (33.3)	
Postoperative complications [*n* (%)]			0.365
Wound infection	1 (9.1)	1 (33.3)	
Adhesive ileus	2 (18.1)	1 (33.3)	

*SR, segmental resection; PA, primary anastomosis*.

Operative management was performed differently in each cases; either a single operation (segmental resection with primary anastomosis or manual detorsion) or a staged operation (segmental resection with ileostomy) but there were no statistical differences. In group A, a staged operation was done in 7 patients (63.6%) and single operation in 4 patients (36.4%). In group B, the 2 operation types were done equally among the patients. In cases of staged operations, the second procedure was performed within 2 months. Although there a few complications were noted after the operation, there was no mortality.

## Discussion

In this study, we considered small intestinal volvulus without malrotation or small intestinal volvulus not associated with other diseases as a PSV, and described its characteristics. However, there might be some confusion with regard to the designation of a segmental volvulus, because several studies reported such cases as a volvulus without malrotation or non-fixation. However, variable conditions causing a segmental volvulus of the small intestine in the absence of malrotation have been reported (8, 11, 12). Thus, this could lead to the misinterpretation of a segmental volvulus of the small intestine due to other diseases as a PSV. Therefore, it seems appropriate to use the term PSV distinctively for abnormal twisting of a bowel loop around the axis of its own mesentery without any other intra-abdominal lesions, instead of describing it as “volvulus without malrotation (or malposition).” On the other hand, “segmental volvulus due to [predisposing disease]” may be used to describe a segmental volvulus due to other predisposing factors.

The etiology of PSV remains unknown. There are several possible conditions that could induce a volvulus, such as a long, narrow, and band-like mesentery; a stasis of meconium or bowel content; rapid change in intra-abdominal pressure; and hyper-peristalsis (2, 8, 11, 14, 15). However, such conditions are not always accompanied by PSV. Thus, a group of diseases with a similar phenotype and different causes may be involved. In our study, a long and narrow mesentery was identified in only 2 cases in group B. Although it may be considered a potential cause of PSV, it could also be a result of repeated torsion and detorsion of the mesentery instead. Drewett et al. suggested that an anatomical predisposition related to long-standing subacute obstruction may be a cause of PSV, especially in preterms (1). A certain disorder of coordinated intestinal motility might lead to an intestinal obstruction at the terminal ileum and induce segmental twisting of the bowel loops. Moreover, meconium in the ileum may also lead to obstruction followed by PSV development (8). Both groups also included preterms; however, no relations between meconium and PSV development was identified. On the other hand, stasis of the meconium, rapid changes in intra-abdominal pressure, and hyper-peristaltic movement have also been postulated as potential causes only in preterms (14, 15). Therefore, none of these could explain the cause of PSV in this study.

The main clinical presentation was non-specific abdominal distention in both groups, although bilious vomiting was commonly observed in neonatal group A. Therefore, it has always been challenging to diagnose a PSV preoperatively, because it shows no typical presentations and there are no reliable methods. Moreover in this study, none of the cases were preoperatively diagnosed as PSV. In case of prenatal diagnosis, fetal bowel dilatation due to ileus and necrosis of the intestinal wall, and hemorrhagic fetal ascites due to perforation of the necrotic bowel have been reported; however, the diagnosis is non-specific (10). In this study, as abnormal antenatal findings were seen only in neonatal group A, it could be assumed that there are different etiologic factors between the 2 groups. Moderate to severe fetal anemia were reported to be suggestive signs of fetal intestinal volvulus (12). Radiographic findings only revealed various degrees of dilated bowel loops in previous studies (2, 11). Moreover, Doppler ultrasonography generally did not reveal the whirl sign, a specific finding of midgut volvulus. On the other hand, a peripheral segmental vascular whirl was occasionally seen in some cases. The common findings were ascites, dilated bowel loop with hypoperistalsis, and bowel wall thickening (8). A contrast study also revealed non-specific findings, such as ileal stenosis, microcolon, or small bowel obstruction, similar to those in the patients of our series (2, 8, 10, 13). A repeat contrast study or computed tomography could aid in making the diagnosis, and a bird-beak appearance in contrast enema, which suggests a knot in the distal intestine, would be a helpful sign (7).

Prompt surgical management is inevitable because a PSV has been known to progress more rapidly to ischemic change of the involved segment than a volvulus associated with a malrotation (2). PSV required a segmental resection in most cases (13 of 14, 92.9%). Contrary to the usual midgut volvulus related to malrotation, the anatomical location of PSV seems to contribute to this presentation (2, 15, 16). When a segmental volvulus occurs, it may lead to constricted volvulus of the involved segment due to a tightly fixed proximal or distal portion, and could result in early vascular compromise with a rapid progression to ischemia. Ultimately, frequent resection of the involved segment would be required. In our series, bowel resection was also needed because of ischemic change or perforation, except for an incidentally found asymptomatic case. Although the difference was not significant, perforation of the involved segment was identified only in neonatal group.

A previous study suggested 2 different clinical types according to onset of clinical presentation, especially in preterms, and divided the patients into the early onset and late onset groups (1). Moreover, in other studies, patients who presented symptoms and were diagnosed after the neonatal period were also described (2, 17), but were not categorized as a separate group.

Similar ratio of preterms between the groups (36.7 vs. 33.3%), presences of a long and narrow mesentery only in the beyond neonatal group and presences of antenatal abnormal sonographic findings, such as bowel dilatation only in the neonatal group would support the differences between two groups. PSV occurred through the whole small intestinal tract associated with a relatively high rate of perforation in neonatal group A and only at the ileum in beyond neonatal group B. These different findings could be caused by different etiologies, and suggest that PSV may be a set of diseases that have similar presentations and different causes.

Additionally, although a segmental resection was performed in most cases regardless of postnatal age at diagnosis, a relatively good result without mortality was obtained.

This study is limited by its small sample size and single institution design. However, in the surgical perspectives, our findings suggest that PSV not only presents different clinical patterns according to postnatal age at diagnosis but may also have favorable clinical courses with prompt surgical management irrespective of age. Considering the rarity of this condition, further studies should be attempted.

## Ethics Statement

This study is a retrospective cohort study. Therefore, it was impossible to get consent from patients and their guardians in advance. This study was not for research purposes about human subjects, so it does not contain sensitive data. The study is being published in an open access journal after collecting data of treatments and operations for the past 10 years. Moreover, the Pusan National University Yangsan Hospital Institutional Review Board approved the progress of this project without informed consent (IRB No. L-2018-138).

## Author Contributions

Y-HC and H-YK contributed to study conception, design and revising manuscript and finalizing submission. S-HK contributed to data analysis and interpretation, and drafting of manuscript.

### Conflict of Interest Statement

The authors declare that the research was conducted in the absence of any commercial or financial relationships that could be construed as a potential conflict of interest.
